# Intra-Lesional Injection of the Novel PKC Activator EBC-46 Rapidly Ablates Tumors in Mouse Models

**DOI:** 10.1371/journal.pone.0108887

**Published:** 2014-10-01

**Authors:** Glen M. Boyle, Marjorie M. A. D'Souza, Carly J. Pierce, Ryan A. Adams, Aaron S. Cantor, Jenny P. Johns, Lidia Maslovskaya, Victoria A. Gordon, Paul W. Reddell, Peter G. Parsons

**Affiliations:** 1 Drug Discovery/Cancer Drug Mechanisms Group, QIMR Berghofer Medical Research Institute, Brisbane, Queensland, Australia; 2 School of Medicine, University of Queensland, Brisbane, Queensland, Australia; 3 Department of Otolaryngology, Head and Neck Surgery, Princess Alexandra Hospital, Brisbane, Queensland, Australia; 4 QBiotics Ltd., Yungaburra, Queensland, Australia; University of Pittsburgh School of Medicine, United States of America

## Abstract

Intra-lesional chemotherapy for treatment of cutaneous malignancies has been used for many decades, allowing higher local drug concentrations and less toxicity than systemic agents. Here we describe a novel diterpene ester, EBC-46, and provide preclinical data supporting its use as an intra-lesional treatment. A single injection of EBC-46 caused rapid inflammation and influx of blood, followed by eschar formation and rapid tumor ablation in a range of syngeneic and xenograft models. EBC-46 induced oxidative burst from purified human polymorphonuclear cells, which was prevented by the Protein Kinase C inhibitor bisindolylmaleimide-1. EBC-46 activated a more specific subset of PKC isoforms (PKC-βI, -βII, -α and -γ) compared to the structurally related phorbol 12-myristate 13-acetate (PMA). Although EBC-46 showed threefold less potency for inhibiting cell growth than PMA *in vitro*, it was more effective for cure of tumors *in vivo*. No viable tumor cells were evident four hours after injection by *ex vivo* culture. Pharmacokinetic profiles from treated mice indicated that EBC-46 was retained preferentially within the tumor, and resulted in significantly greater local responses (erythema, oedema) following intra-lesional injection compared with injection into normal skin. The efficacy of EBC-46 was reduced by co-injection with bisindolylmaleimide-1. Loss of vascular integrity following treatment was demonstrated by an increased permeability of endothelial cell monolayers *in vitro* and by CD31 immunostaining of treated tumors *in vivo*. Our results demonstrate that a single intra-lesional injection of EBC-46 causes PKC-dependent hemorrhagic necrosis, rapid tumor cell death and ultimate cure of solid tumors in pre-clinical models of cancer.

## Introduction

Surgical excision and ionizing radiation of affected sites have been the mainstay for treatment of cancer patients for decades. Whilst often effective, efficacy of these treatments can be limited by various factors including the condition of the patient, the proximity of adjacent vital tissues, inaccessibility of the tumor and intolerance of normal tissue for repeated courses of treatment. In some of these cases, intra-tumoral treatment may be more appropriate, particularly when surgical intervention is not possible. There have therefore been many attempts to deliver localized therapies, such as injection of anti-cancer agents [1]–[3] and lethal implants [4], aiming for palliation or even cure. Intra-tumoral treatment may have the advantage of allowing for much higher drug concentrations at the tumor site, and potentially less toxicity than systemic agents. However, a limiting factor for greater use of intra-tumoral treatments appears to be lack of suitable agents rather than delivery technologies.

The protein kinase C (PKC) family are ubiquitous serine-threonine kinases found in many cell types that translocate to membranes after activation and regulate diverse downstream processes, like proliferation, apoptosis, differentiation, and migration. There are ten main human isoforms that comprise three subgroups divided according to sequence homology and cofactor requirements. The classical PKC subgroup includes -α, -βI and-βII (alternatively spliced from the same gene), and -γ, which require binding of calcium and DAG to activate the enzyme. Members of the novel PKC subgroup include -δ, -ε, -η and -θ, which require DAG for activation but are calcium-independent. The atypical PKCs, -ι and -ζ, are independent of calcium and DAG, but have been shown to be activated by distinct lipids and protein-protein interactions [5], [6].

Inhibition of PKC signaling has been targeted as an anti-cancer treatment as PKC isozymes are known to play roles in cellular proliferation and vasculature formation [7]–[9], important for tumor growth. However, several clinical trials performed with compounds thought to inhibit PKC signaling have had disappointing results [9]. For example, enzastaurin, an orally available specific inhibitor of PKC-β, showed limited efficacy as a single agent in Phase II studies of advanced diffuse large B-cell lymphoma or non-small cell lung cancer [10], [11]. Clinical trials of enzastaurin with additional chemotherapy agents are underway.

In contrast, previous work has shown that intravenous administration of the prototypic PKC activating compound phorbol 12-myristate 13-acetate (PMA) to patients suffering from myleocytic malignancies resistant to chemotherapy resulted in remission [12], [13]. We have previously demonstrated that three topical applications of the PKC-activating ingenol ester PEP005 (ingenol mebutate or ingenol 3-angelate) are sufficient for enduring regression of skin cancer lesions (including melanoma) in pre-clinical models [14], [15]. PEP005 has recently been approved for topical treatment of actinic keratoses [16], [17], and shows efficacy in additional models of squamous cell carcinoma [18], [19]. We now describe a novel PKC-activating compound, EBC-46, and demonstrate that a single intra-lesional injection is sufficient for enduring regression and ultimate cure of diverse tumor types in pre-clinical models of cancer.

## Materials and Methods

### Reagents

EBC-46 was purified from the kernels of the fruit from *Fontainea picrosperma*, was provided by QBiotics Ltd. (Yungaburra, Queensland) at greater than 97% purity. Phorbol 12-myristate 13-acetate (PMA), dihydroethidium and bisindolylmaleimide-1 (BIS-1) were purchased from Sigma (St. Louis, MO).

### Cell lines

All cell lines used in this study were purchased from ATCC (SK-MEL-28, B16-F0, HeLa, FaDu, MCF-7, HT-29) except MC-38 [20], [21] and MM649 [22] cells, which were described previously. Cell lines were grown in complete media (RPMI-1640 supplemented with 10% heat-inactivated fetal calf serum, 3 mM HEPES, 100 U/ml penicillin and 100 µg/ml streptomycin (CSL Ltd, Melbourne, Australia)). Cells were routinely checked by STR profiling, and for mycoplasma infection by Hoechst staining [23] and PCR, and were always negative.

### Human ethics statement

This study was performed in strict accordance with the recommendations in the Australian National Statement on Ethical Conduct in Human Research (2007 - Updated December 2013), of the National Health and Medical Research Council of Australia. All protocols were reviewed and approved by the QIMR Berghofer Medical Research Institute Human Research Ethics Committee (QIMR-HREC), approval number P764. All participants provided written informed consent to donate their blood samples for research.

### Measurement of reactive oxygen species production in PMN cells

PMN cells were isolated from peripheral blood from healthy volunteers. Aliquots of 2×10^6^ PMN cells were stained incubated for 15 min at 37°C with 10 µg/ml dihydroethidium (DHE). 2×10^6^ unstained PMN cells were incubated at 37°C to act as an unstained control. Where appropriate, PMN cells were also incubated with 1 µM bisindolylmaleimide-1. The stained PMN cells were treated with the either PMA or EBC-46 for 15 min, and the change in fluorescence resulting from oxidation of DHE to ethidium bromide measured on a dual beam FACSCalibur (BD Biosciences, Franklin Lakes, NJ) using CellQuest Pro (BD Biosciences) software.

### PKC-EGFP translocation

pPKC-α, -βII, -γ, -θ and –ζ-EGFP were purchased from Clontech (Moutain View, CA). pPKC-βI, -δ, -ε, -η and -ι-EGFP were constructed in-house. PKC isoforms were cloned from Universal Human Tumor cDNA (Life Technologies, Carlsbad, CA) and ligated into pPKC-ζ-EGFP digested with *Xho*I/*Sac*II. The identity and fidelity of all PKC isoforms was verified by Sanger sequencing. 96-well plates were seeded with 3×10^4^ cells/well of SK-MEL-28 in complete media, and incubated at 37°C, 5% CO_2_, and 95% humidity. After 24 h, cells were transiently transfected with a 1∶3 ratio of pPKC-EGFP vector DNA: Lipofectamine 2000 (Life Technologies) (0.16 µg DNA: 0.48 µl Lipofectamine per well) in Opti-MEM media (Life Technologies). After 24 h incubation, cells were washed with phosphate-buffered saline (PBS) and treated with 100 ng/ml of either PMA or EBC-46 for 1 h. Cells were washed twice with ice-cold PBS, fixed with 2% formaldehyde/0.2% gluteraldehyde in PBS, washed twice again with ice-cold PBS, and overlaid with 100 µl of PBS. Fluorescent cells were examined with an AMG EvosFl (Life Technologies) inverted fluorescence microscope at 40×. Micrographs were taken such that at least 50 fluorescent cells/well were captured, and transmitted light images were overlaid with fluorescence images for control wells to calculate transfection efficiency. Cells were counted in each image using ImageJ (NIH) and classified according to the localization of fluorescence as cytoplasmic, plasma membrane, perinuclear membrane, or other membrane (mitochondria, endoplasmic reticulum, Golgi apparatus, or unknown). A total of three independent experiments were performed for each PKC isoform.

### Immunofluorescence

HeLa cells were treated with either 175 nM (100 ng/ml) PMA or EBC-46, or vehicle alone for 1 h. PKC-α (Cat. No. 2056; Cell Signaling Technologies) was detected by immunofluorescence as described by the manufacturer. Images were acquired using a Leica TCS Inverted Fluoresence Microscope with a Nikon DS-Fi1C camera.

### PKC kinase assay

HeLa cells were treated with 175 nM (100 ng/ml) or 17.5 µM (10 µg/ml) of either PMA or EBC-46 for 1 h. Cells were lysed and stored at −80°C, before the level of PKC-specific kinase activity was measured in 30 µg HeLa lysate using the PKC Kinase Activity Assay Kit (AbCam, Cambridge, U.K.; Cat. No. ab139437) as described by the manufacturer. Assays were performed in triplicate with the mean ± SD shown.

### Cell growth assay

Cells were seeded at sub-confluence (5,000 cells/well) in 96-well microtitre plates. All drugs and inhibitors were diluted in complete media. Controls were treated with vehicle alone. Cells were treated with PMA or EBC-46 the day after seeding and cultured for 4 days. To measure inhibition of cell growth, attached cell lines were assayed using sulforhodamine B (SRB) [24], [25]. The experiments were repeated at least twice and the mean ± SD was determined in Prism 6 (GraphPad Software, San Diego, CA).

### Animal ethics statement

This study was performed in strict accordance with the recommendations in the Australian Code for the Care and Use of Animals for Scientific Purposes 8th Edition (2013), of the National Health and Medical Research Council of Australia. All protocols were reviewed and approved by the QIMR Berghofer Medical Research Institute Animal Ethics Committee (QIMR-AEC), approval numbers A0106-042M and A0404-606M. All mice were housed in a specific pathogen free (SPF) facility, with 12 hours light/dark cycle and continual access to food and water. All mice were monitored daily and tumor volume measured at least twice weekly, recorded using digital calipers and expressed as mm^3^ according to the formula A×b×b×0.5 where A the length and b the measured breadth of the tumor. Mice were also assessed for clinical signs according to a QIMR-AEC approved clinical score sheet for distress during the period of the experiment to determine whether the treatments (i.e. tumor burden and effects of drugs) were causing distress to the mice to a degree and to where they should be euthanased (Table 1). Scores for each parameter were summed to give a possible total of 8. Less than 3 was considered a mild clinical score, between 3 and 6 was considered a moderate clinical score, and over 6 was considered a severe clinical score. The experiment was ceased when an unacceptable clinical score (>6) was reached, or the cumulative tumor burden of the mouse exceeded 1,000 mm^3^. Mice were humanly euthanized by asphyxiation at the end of the experiment.

**Table 1:** Clinical scoring for Drug Treated and Tumor Bearing Mice.

Criteria	Grade 0	Grade 1	Grade 2
Weight loss	<10%	>10 to <25%	>25%
Posture	Normal	Hunching noted only at rest	Severe hunching impairs movement
Activity	Normal	Mild to moderately decreased	Stationary unless stimulated
Fur texture	Normal	Mild to moderate ruffling	Severe ruffling/poor grooming

### EBC-46 treatment of tumors in mice

SK-MEL-28, MM649, FaDu (2×10^6^) or B16-F0 (1×10^5^) cells were injected (two tumors per mouse) on the hindquarter of 5 week old immunocompromised BALB/c *Foxn1^nu^* mice or C57BL/6J mice. When the tumors reached approximately 50 mm^3^ (SK-MEL-28 and MM649) or 100 mm^3^ (FaDu and B16-F0), mice in the control group were treated with vehicle (20% propylene glycol in water, 50 µl), and the treatment group received 50 nmol (30 µg) EBC-46 in vehicle, via a single intra-tumoral injection. Mice were euthanized when the cumulative tumor burden per mouse exceeded 1,000 mm^3^ or at the end of the experiment.

### Pharmacokinetic study of EBC-46 in tumor and non-tumor-bearing mice

Nine BALB/c *Foxn1^nu^* mice were injected with 2×10^6^ MM649 melanoma cells, one tumor per mouse. Tumors were monitored until they reached approximately 100 mm^3^. Mice were then treated by injecting 50 nmol (30 µg) EBC-46 either into the tumor (tumor bearing mice) or into normal skin (sub-cutaneously, 9 tumor-free mice). Blood (maximum of 150 µl) was collected from the tail vein by nicking at the base of the tail at 30 min, 1, 2, 4, 8 and 24 h post-treatment (3 animals at 30 min and 4 h, 3 animals at 1 and 8 h, 3 animals at 4 and 24 h) into a lithium heparin Microvette CB300 blood collection system (Sarstedt, Numbrecht, Germany), and processed to plasma by centrifugation at 2,000 *g* for 5 min at 20°C until separation occurred. Plasma was frozen at −80°C until analysed. Samples were analyzed using a specifically developed HPLC method to detect EBC-46 in mouse serum against a spiked standard curve. Erythema and oedema were rated using a five point scale (0 to 4; none to severe) 24 h after injection. Weight of animals was determined immediately prior to, and 24 h following treatment.

### *Ex vivo* analysis of tumor cell survival

SK-MEL-28 or FaDu cells were injected (two tumors per mouse) on the hindquarter of 5 week old immunocompromised BALB/c *Foxn1^nu^* mice. When the tumors reached approximately 100 mm^3^, mice in the control group were treated with 20% propylene glycol in water, and the treatment group received 50 nmol (30 µg) EBC-46 via a single intra-tumoral injection. Mice were euthanized at time of injection, 1, 2, 4, 8 and 24 h post-treatment with vehicle or EBC-46, and tumors were harvested. Tumors were dissected, briefly dissociated with collagenase A, and finally resuspended in culture medium. Serial 3-fold dilutions of the cell suspension were cultured *in vitro* for 6 days, and the SRB assay used to compare the growth of viable EBC-46-treated tumor cells with that of vehicle treated controls.

### EBC-46 treatment in neutrophil-depleted mice

SK-MEL-28 cells (2×10^6^) were injected (two tumor sites per mouse) into the flanks of thirty 5- to 6-week old male BALB/c *Foxn1^nu^* mice (*n* = 5 mice and *n* = 10 tumors/group). When tumors had reached >50 mm^3^, ten mice were given i.p. injections of rat anti-neutrophil targeting antibody mLy-6G (100 µg in PBS; clone 1A8, Cat. # BE0075-1) or rat IgG2a isotype control (clone 2A3, Cat. # BE0089) from BioXCell (West Lebanon, NH). The antibodies were injected on days −2, 0, and 2, relative to initiation of vehicle or EBC-46 (25 nmol, 15 µg) treatment on day 0. An additional ten control mice received no antibody. The tumors on a total of 15 mice (5 each of no antibody, IgG2a isotype control or anti-mLy-6G antibody) were treated with 25 nmol EBC-46 per site (15 µg in 20% propylene glycol in water at 300 µg/ml, 50 µl injection), while the tumors on the remaining 15 mice were treated with 50 µl 20% propylene glycol in water only. Blood was taken from tail tips on Days −2, 0 and 2, and then twice weekly, smeared, and air dried on glass slides before being stained with Quick Dip (Fronine Laboratory Supplies, Rivertone, NSW, Australia). Tumor size was measured with calipers twice weekly. Mice were euthanized when the cumulative tumor burden per mouse exceeded 1,000 mm^3^ or at the end of the experiment.

### *In vitro* permeability assay

HUVEC cells (Invitrogen/Life Technologies) were grown as described by the manufacturer and used at passage 4 to 6. Media and supplements (M200 [Cat. No. M200PRF500] and Low Serum Growth Supplement [Cat. No. S-003-10] respectively, Life Technologies) were prepared as directed. The *In vitro* Vascular Permeability Kit was from Millipore (Billerica, MA; Cat. No. ECM642). All assays were performed as described by the manufacturer. Assays were performed in at least triplicate wells.

## Results

### EBC-46 is a novel Protein Kinase C-activating compound

**Figure 1:**
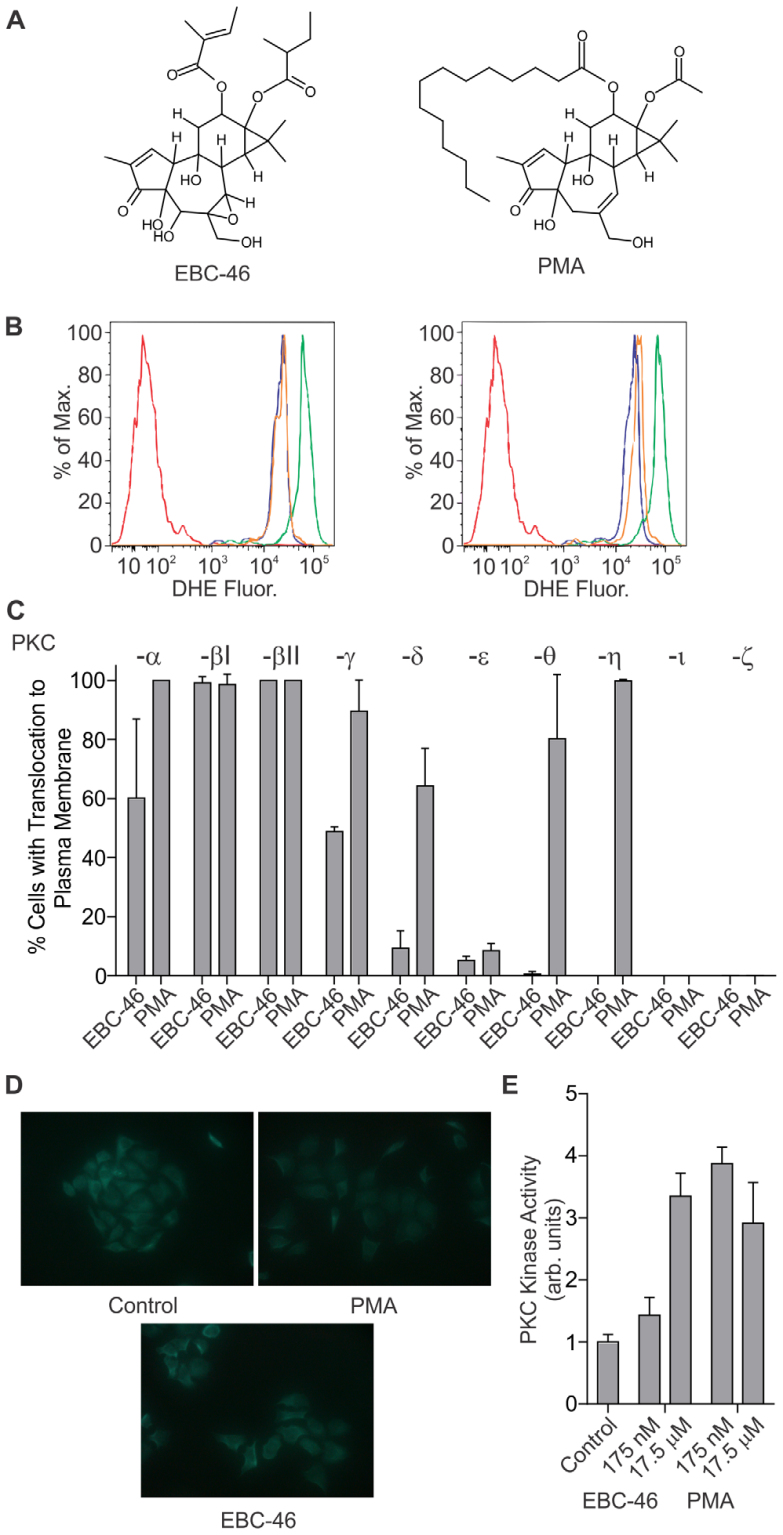
EBC-46 is a novel Protein Kinase C-activating compound. **A**. The structure of EBC-46. Structure of PMA shown as a comparison. **B**. Production of reactive oxygen species following treatment of PMN cells. PMN cells were pre-loaded with dihydroethidium bromide and pre-treated where indicated with 1 µM bisindolylmaleimide-l for 15 min, then stimulated with 175 nM (100 ng/ml) of either EBC-46 (left) or PMA (right) for 15 min at 37°C. Untreated PMN cells – red, PMN cells pre-loaded – blue, loaded PMN cells treated with either 175 nM (100 ng/ml) PMA or EBC-46 – green, loaded PMN cells treated with either 175 nM (100 ng/ml) PMA or EBC-46 in presence of bisindolylmaleimide-l – yellow. Data is representative of three independent experiments. **C**. PKC-EGFP isoform translocation in transiently transfected HeLa cells following 1 h treatment with either 175 nM (100 ng/ml) PMA or EBC-46. Data is from assessment of at least 50 cells per well from each of triplicate transient transfection experiments. Error bars – SD. **D**. HeLa cells were treated with either 175 nM (100 ng/ml) EBC-46 or PMA for 1 h, or vehicle alone (Control). PKC-α was detected by immunofluorescence. **E**. PKC kinase assay. HeLa cells were treated with the indicated amounts of EBC-46 or PMA for 1 h. A total of 30 µg of lysate was used to determine PKC-specific kinase activity. Assays were performed in triplicate (n = 3) with the mean ± SD shown.

**EBC-46.** (12-Tigloyl-13-(2-methylbutanoyl)-6,7-epoxy-4,5,9,12,13,20-hexahydroxy-1-tigliaen-3-one; C_30_H_42_O_10_; 562.65 g/mol) is a novel compound purified from a commercially-sustainable natural source. It is structurally similar to the prototypic PKC-activating compound phorbol 12-myristate 13-acetate (PMA), but considerably less hydrophobic due to short ester side-chains and hydroxylation in the B ring (Figure 1A). To investigate if EBC-46 activated PKC, we initially examined the production of reactive oxygen species following treatment of PMN cells. The induction of oxidative burst in human PMN by PKC activators has been previously described [26], [27]. Treatment of PMN cells with 175 nM (100 ng/ml) PMA lead to an increase in fluorescent signal that corresponded with the oxidation of dihydroethidium bromide to fluorescent ethidium bromide. Pre-treatment with 1 µM bisindolylmaleimide-l (pan-PKC inhibitor) prevented this increase in fluorescent signal (Figure 1B, right) indicating PKC-activation dependence. Similarly, treatment of PMN cells with 175 nM (100 ng/ml) EBC-46 also led to an increase in fluorescent signal by oxidation, that was prevented by pre-treatment with bisindolylmaleimide-l (Figure 1B, left).

Transient transfection of HeLa cells with PKC isoforms tagged to EGFP and subsequent treatment with PMA resulted in efficient translocation to the plasma membrane of –α, -βI, βII, -γ, -δ, -θ, and –η subtypes (Figure 1C). Modest translocation of PKC-ε was observed following 1 h treatment, consistent with previous results from others [28]. As expected, no translocation was seen for the atypical PKC-ι or -ζ isoforms lacking a C1 DAG-binding domain after treatment with PMA. Treatment with EBC-46 resulted in high percentage of cells showing translocation of PKC-βI and –βII isoforms, with less translocation seen for the –α, –γ, –δ, and -ε isoforms. No translocation of the atypical PKC-ι or -ζ isoforms was observed following EBC-46 treatment. Interestingly, EBC-46 treatment did not result in significant translocation of the –θ and –η isoforms to the plasma membrane suggesting that PMA and EBC-46 differ in their selectivity and/or potency towards specific PKC isoforms. These results were confirmed in SK-MEL-28 cells (Figure S1). However, transient transfection of PKC-ε or -ι isoforms was toxic to SK-MEL-28 cells, while transfection of PKC-δ localized to the mitochondrial membrane and treatment with PMA or EBC-46 had no effect (Figure S1). EBC-46 treatment resulted in the translocation of endogenous PKC-α to the plasma membrane in HeLa cells, similar to that with PMA (Figure 1D). Further, an *in vitro* kinase assay demonstrated an increase of PKC kinase activity in HeLa cells treated with EBC-46, particularly at 17.5 µM (10 µg/ml) (Figure 1E). Taken together, these results suggest that EBC-46 is a novel activator of PKC, in particular of the –β isoforms.

### EBC-46 is efficacious *in vivo*, independent of tumor cell sensitivity *in vitro*

**Figure 2:**
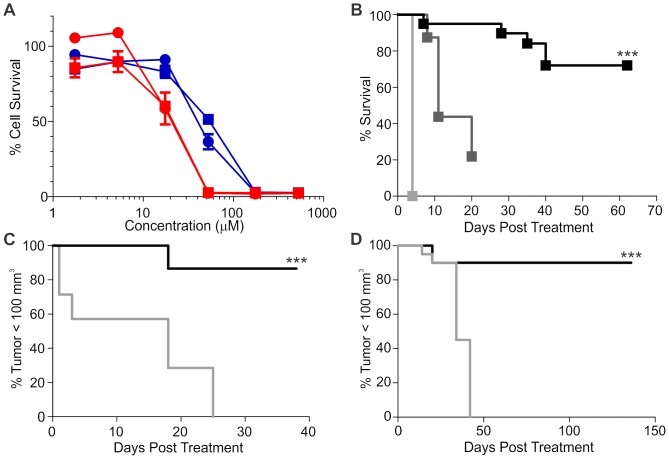
EBC-46 efficacy *in vivo* is independent of tumor cell sensitivity *in vitro*. **A**. Dose response for cell killing by EBC-46 compared to PMA. B16-F0 (circles) or SK-MEL-28 (squares) melanoma cells were treated with the indicated doses of either EBC-46 (blue) or PMA (red) for 4 days, before assay for cell survival using the sulforhodamine B assay. Data shown are mean ± SD from triplicate readings from three independent experiments, n = 3. **B**. Kaplan Meier analysis of survival of C57BL/6J mice with B16-F0 tumors. Mice with two tumors were treated with single bolus doses of vehicle alone (20% propylene glycol in water; light grey), 50 nmol (30 µg) PMA (mid grey) or 50 nmol (30 µg) EBC-46 (black). Mice were euthanized once the total tumor volume reached 1,000 mm^3^ per animal. Squares – censored data. Difference between survival following treatment with EBC-46 or PMA was significant (*** p = 0.0004; Log-rank (Mantel-Cox) Test). Data was obtained from 6 mice per group, 2 tumors per mouse; n = 12. **C**. Kaplan Meier analysis of SK-MEL-28 melanoma tumors reaching >100 mm^3^ in BALB/c *Foxn1^nu^* mice following single treatment with vehicle alone (grey) or 30 µg EBC-46 (black) (***, p<0.0001; Log-rank (Mantel-Cox) Test). Data was obtained from 5 mice per group, 2 tumors per mouse; n = 10. **D**. Kaplan Meier analysis of MM649 melanoma tumors reaching >100 mm^3^ in BALB/c *Foxn1^nu^* mice following single treatment with vehicle alone (grey) or 50 nmol (30 µg) EBC-46 (black) (***, p<0.0001; Log-rank (Mantel-Cox) Test). Data was obtained from 5 mice per group, 2 tumors per mouse; n = 10.

Previous studies have shown activators of PKC to induce cell death in cancer cells *in vitro* and *in vivo* [14]. We therefore tested clonogenic-type cell survival of EBC-46 in comparison to PMA. Treatment with PMA for 4 days led to almost complete cell death at 52.5 µM for both B16-F0 and SK-MEL-28 cells, with the 50% lethal dose (LD_50_) of approximately 17.5 µM for each cell line (17.63±0.13 and 17.81±0.15 µM respectively). In comparison, EBC-46 was less potent for cell killing than PMA *in vitro* (Figure 2A). Treatment with EBC-46 for the same period led to complete cell killing at 175 µM, with an LD_50_ of approximately 52.5 µM for each cell line (B16-F0, 52.25±0.11; SK-MEL-28, 52.60±0.18 µM). Similar results were obtained for a panel of cell lines, including HeLa, FaDu, HT-29, MCF-7, MM649 and MC-38 (Figure S2); in each case EBC-46 was less potent for cell killing than PMA *in vitro*. However, these results show that EBC-46 has a direct effect on cell survival *in vitro*.

We tested the *in vivo* efficacy of both PMA and EBC-46 in the B16-F0/C57BL/6J mouse melanoma model via intra-tumoral injection. Each mouse was injected with 500,000 B16-F0 cells per flank, and the tumors allowed to reach 100 mm^3^. Intra-tumoral injection of vehicle alone (20% propylene glycol in water) into established tumors had no effect, with all mice reaching the maximal tumor burden 4 days after treatment (Figure 2B). Intra-lesional injection of 50 nmol (30 µg) PMA in vehicle delayed tumor growth compared to vehicle alone, with all mice reaching maximal tumor burden 20 days post-treatment. Importantly, all treated sites relapsed. In contrast, intra-lesional treatment with 50 nmol (30 µg) EBC-46 led to an initial swelling of the tumor site followed by rapid ablation of the tumors. Treatment with a single bolus injection of 50 nmol (30 µg) EBC-46 showed a significant (p = 0.0004, Log-rank (Mantel-Cox) test) extension of time to euthanasia due to tumor burden compared to treatment with PMA, indicating anti-tumor efficacy. There was more than 70% total cure in the EBC-46 treatment group that was censored in the data due to single sites recurring of the two treated per mouse (15 of 20; 75% cured).

We further evaluated the anti-tumor efficacy of EBC-46 in additional mouse models of cancer. Intra-lesional treatment of BALB/c *Foxn1^nu^* mice, xenografted with SK-MEL-28 or MM649 human melanoma tumors (>50 mm^3^), with 50 nmol (30 µg) EBC-46 again led to an initial swelling of the tumor treatment site followed by ablation of the tumors compared to treatment with vehicle alone (20% propylene glycol in water). There were three recurrences after EBC-46 treatment of SK-MEL-28 tumors within the 40 day observation period (2>100 mm^3^, 1<100 mm^3^), and a single recurrence of a MM649 tumor within the 150 day observation period (>100 mm^3^) (Figure 2C and D respectively). Differences in tumor recurrence was highly significant for both SK-MEL-28 and MM649 (p<0.0001, Log-rank (Mantel-Cox) Test). In the MM649 tumor model, all other sites treated with EBC-46 in this study remained tumor free with no recurrence for 12 months (data not shown). Additional models showed efficacy against head and neck cancer (FaDu, Figure S3 and S4A) and colon cancer (HT-29, Figure S4B; MC-38, Figure S4C). These results suggest that a single bolus intra-lesional treatment with EBC-46 can lead to an enduring ablation of tumor cell growth *in vivo*.

### Pharmacokinetic studies suggest EBC-46 remains at the tumor injection site

**Figure 3:**
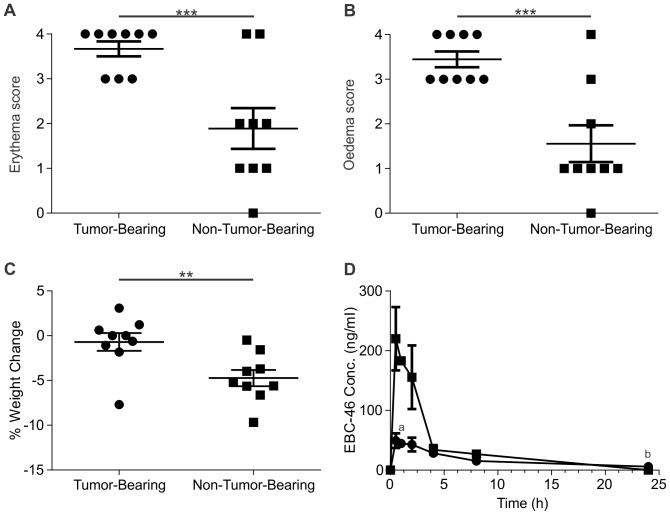
EBC-46 treatment induces greater effects when injected into tumors compared to normal skin. EBC-46 was injected into male BALB/c *Foxn1^nu^* mice either bearing (n = 9) or not bearing (n = 9) tumors, as a single intra-lesional or sub-cutaneous injection, respectively. **A**. Erythema at injection site 24 hours following treatment with 50 nmol (30 µg) EBC-46 in tumor- or non-tumor-bearing mice. **B**. Oedema at injection site 24 hours following treatment with 50 nmol (30 µg) EBC-46 in tumor- or non-tumor-bearing mice. **C**. Percentage weight change 24 hours following treatment with 50 nmol (30 µg) EBC-46 in tumor- or non-tumor-bearing mice. **D**. Concentration in serum following treatment with 50 nmol (30 µg) EBC-46 in tumor- or non-tumor-bearing mice. Data is from serum from three animals unless otherwise indicated. Error bars – SEM. (**, p<0.01; ***, p<0.005; t-test). a - single data point from EBC-46-treated animals. b - below lower limit of detection of the assay, set at 0.01 ng/ml.

We wished to determine the pharmacokinetic profile of EBC-46 following a single intra-tumoral or sub-cutaneous injection into mice either bearing or not bearing tumors, respectively. Intra-tumoral treatment with EBC-46 of BALB/c *Foxn1^nu^* mice xenografted with MM649 melanoma cells led to significant erythema and oedema of the injection sites, as assessed 24 hours following injection. Sub-cutaneous treatment of normal skin to mimic intra-tumoral treatment (non-tumor bearing mice) led to significantly less erythema (p = 0.0021, t-test; Figure 3A) and oedema (p = 0.0007, t-test; Figure 3B), as assessed 24 hours after treatment. Mice without tumors that were treated sub-cutaneously with 50 nmol (30 µg) EBC-46 lost significantly more weight in 24 h (4.73±0.91% loss) than those mice treated by intra-tumoral injection with EBC-46 (0.70±0.99% loss; p = 0.0087, t-test; Figure 3C). Additionally, blood was collected following administration of EBC-46 to mice, and a quantitative HPLC assay used to determine the level of EBC-46 in the plasma. Results show that less EBC-46 was detected in serum from tumor-bearing mice treated by intra-lesional injection compared to non-tumor bearing mice treated by sub-cutaneous injection (Figure 3D), suggesting that the compound is retained at the tumor site.

### Neutrophils have a minor role in EBC-46 anti-cancer activity

**Figure 4:**
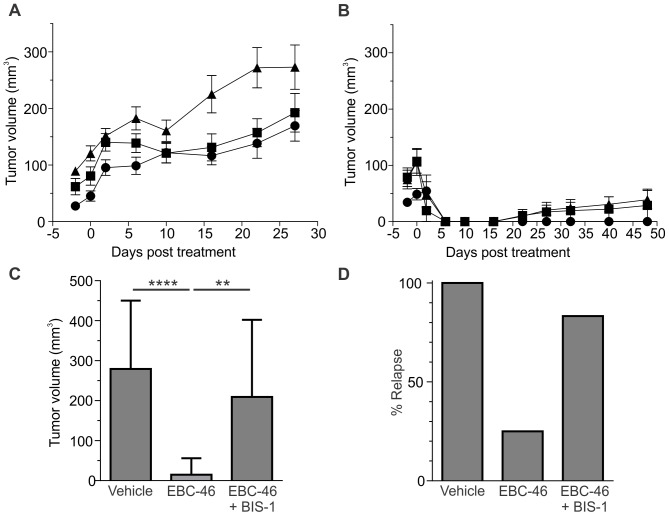
EBC-46 anti-cancer efficacy is PKC-dependent. **A**. The effect of neutrophil depletion on EBC-46 treatment of SK-MEL-28 tumors grown on BALB/c *Foxn1^nu^* mice. Tumor growth in mice given anti-Ly-6G antibody, isotype control antibody or no antibody. SK-MEL-28 cells were injected sub-cutaneously (n = 10 tumors/group; 2 tumors/mouse, 5 mice/group) and allowed to reach approximately 100 mm^3^. Mice were injected with anti-Ly-6G antibody (clone 1A8; 100 µg i.p, on days −2, 0, and 2), with isotype control antibody (IgG2a, clone 2A3; 100 µg i.p, on days −2, 0, and 2) or with nothing. Tumors were treated by intra-lesional injection of vehicle (50 µl of 20% propylene glycol in water) on day 0. The tumor volumes represent the mean volume of individual tumors. • – no antibody, ▪ – control IgG2a antibody, ▴ – anti-mLy-6G antibody. **B**. Tumor growth in mice given anti-Ly-6G antibody, isotype control antibody or no antibody after EBC-46 treatment. As for A, SK-MEL-28 tumors (approximately 100 mm^3^) were treated by intra-lesional injection with 25 nmol (15 µg) EBC-46 (in 50 µl of 20% propylene glycol in water) on day 0. • – no antibody, ▪ – control IgG2a antibody, ▴ – anti-mLy-6G antibody. Error bars – SD. **C**. 500,000 B16-F0 cells were injected into BALB/c *Foxn1^nu^* mice, and allowed to reach >50 mm^3^. Tumors were then treated with either 50 µl vehicle (20% propylene glycol in water), 16.7 nmol (10 µg) EBC-46 in vehicle or 16.7 nmol (10 µg) EBC-46 after pre-treatment with 5 µM bisindolylmaleimide-1 (BIS-1) in vehicle. Tumor size was measured 8 days after treatment. Error bars  =  SD, n = 12. (****, p<0.0001; **, p = 0.0024; t-test). **D**. Number of tumors (as percentage) present 8 days after treatment. Total tumor number n = 12 for each group.

Previous studies have outlined the role of neutrophils in anti-tumor efficacy of PKC agonists [15], [29]. The importance of neutrophils for the anti-cancer activity of EBC-46 was investigated by using an antibody (anti-mLy-6G, clone 1A8) to deplete these cells in mice bearing SK-MEL-28 tumors prior to treatment. SK-MEL-28 tumors were allowed to grow to approximately 100 mm^3^ prior to injection of the antibodies on days −2, 0 and 2 relative to treatment with EBC-46 or vehicle alone. The percentage of neutrophils in WBC counts in peripheral blood were determined for each of the groups. Following treatment with anti-Ly-6G antibody, the percentage of neutrophils in the peripheral blood fell from 62% of total leukocytes to 6% after a single treatment (Day 0), while the isotype control antibody had no effect (data not shown). Tumors in mice receiving the anti-Ly-6G neutrophil antibody grew slightly more rapidly compared to SK-MEL-28 tumors receiving either no-antibody or isotype control (IgG2a) antibody following a single treatment with bolus vehicle (Figure 4A), consistent with previous observations [15]. Ablation of the SK-MEL-28 tumors following treatment with 25 nmol (15 µg) EBC-46 (a lower dose of EBC-46 was used to reduce efficacy) was apparent in all groups (Figure 4B). There were no tumor recurrences in the group treated with EBC-46 alone in the 48 day observation period. However, we observed 4 recurrences in the mice receiving the anti-Ly-6G antibody compared to a single recurrence in the mice treated with the isotype control (IgG2a) antibody (Figure 4B). Although these data were not statistically significant (p = 0.30, Fisher's exact test), these results may suggest that neutrophils play only a minor role in the anti-tumor efficacy of EBC-46 in this model.

### EBC-46 anti-cancer efficacy is PKC-dependent

We wished to assess if PKC activation by EBC-46 was necessary for the anti-cancer efficacy of the compound. We therefore tested the *in vivo* efficacy of EBC-46 in BALB/c *Foxn1^nu^* mice xenografted with B16-F0 mouse melanoma cells via intra-tumoral injection in the presence or absence of bisindolylmaleimide-1, a PKC inhibitor. Each mouse was injected with 500,000 B16-F0 cells per injection site, and the tumors allowed to reach >50 mm^3^. Intra-tumoral injection of vehicle alone (20% propylene glycol in water) into established tumors had no effect, with all tumors continuing to increase in size over the following 8 days (Figure 4C). Intra-lesional injection of 16.7 nmol (10 µg) EBC-46 (lower dose to reduce efficacy) in vehicle led to a rapid ablation of the tumors (p<0.0001, t-test, compared to vehicle treated sites; Figure 4C), with only 25% of treated sites showing tumor presence 8 days after treatment (9 of 12 sites with no tumor, Figure 4D). In contrast, intra-lesional treatment with 16.7 nmol (10 µg) EBC-46 after pre-treatment with 5 µM bisindolylmaleimide-1 for 1 h, resulted in the majority of tumors continuing to grow (p = 0.0024, t-test compared to EBC-46 alone treated sites; Figure 4C), with 83.3% of treated sites showing the presence of tumors (2 of 12 sites with no tumor, Figure 4D). Co-injection of EBC-46 with a PKC inhibitor significantly reduced the efficacy of treatment, indicating that the anti-cancer efficacy of EBC-46 is PKC-dependent.

### Treatment with EBC-46 results in rapid loss of tumor cell viability *in vivo*

**Figure 5:**
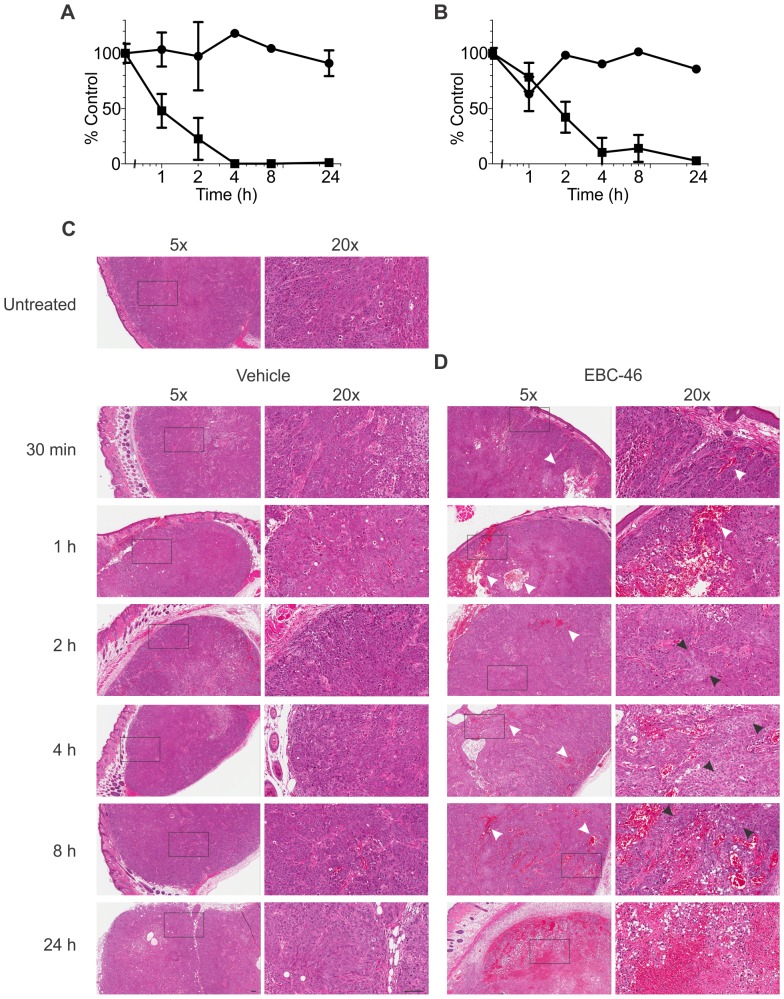
EBC-46 kills tumor cells rapidly *in vivo*. Survival of **A**. SK-MEL-28 melanoma or **B**. FaDu head and neck cancer cells *ex vivo* following treatment with vehicle (20% propylene glycol in water) (circles) or 50 nmol (30 µg) EBC-46 in vehicle (squares). Error bars – SD. Data n = 3 independent experiments. **C**. Histological analysis of FaDu head and neck cancer tumors treated with EBC-46. Representative photomicrographs are shown of the histology tumor sites after intra-lesional injection with vehicle (20% propylene glycol in water) or 50 nmol (30 µg) EBC-46. FaDu tumors were allowed to reach 100 mm^3^ before they were treated with either vehicle or 50 nmol (30 µg) EBC-46, and harvested at the indicated times. White arrows – representative areas of red cell extravasation; black arrows – representative areas of shrunken and pale nuclei. Scale bars  = 100 µm.

Treatment with EBC-46 led to a dramatic ablation of tumors following intra-lesional injection. We therefore wished to examine the timing of loss of viability of tumor cells following treatment with EBC-46 in an *ex vivo* assay. Two tumor models were used, namely SK-MEL-28 and FaDu human tumor cells, in BALB/c *Foxn1^nu^* mice. Tumors were allowed to reach approximately 100 mm^3^ before treatment with 50 nmol (30 µg) EBC-46 or vehicle (50 µl 20% propylene glycol in water), and then removed, dissociated into single cell suspension and assayed for clonogenic growth *in vitro*. The results show that the tumor cells had greatly reduced viability 4 hours after treatment with EBC-46. The results were similar for both SK-MEL-28 (Figure 5A) and FaDu (Figure 5B) tumors. In contrast, tumors treated with vehicle alone remained fully viable. These results suggest that tumor cell viability is compromised following treatment with 50 nmol (30 µg) EBC-46 after as little as 2 to 4 h.

As indicated above, intra-lesional treatment with EBC-46 led to a rapid swelling of the tumor site. We therefore examined FaDu human head and neck cancer tumors xenografted into BALB/c *Foxn1^nu^* mice after treatment with EBC-46 or vehicle alone by histology. Intra-lesional injection of EBC-46 into FaDu tumors also resulted in red cell extravasation within the tumor 1 to 2 h following treatment (Figure 5D). We also observed that tumor nuclei became pale and shrunken 2 to 4 h after treatment, and the tumor cells disorganized 4 to 8 h following EBC-46 treatment (Figure 5D), mirroring the loss of viability seen in the *ex vivo* measurement of efficacy (Figure 5A and B). Similar to other PKC agonists, treatment of normal skin with EBC-46 led to an apparent dilation of smaller blood vessels, and some red cell extravasation in the dermis and subcutis of the skin up to 8 h following treatment (Figure S5B). However, there was no evidence of remaining red cell extravasation 24 h after treatment.

### EBC-46 causes permeability of endothelial cells within tumors

**Figure 6:**
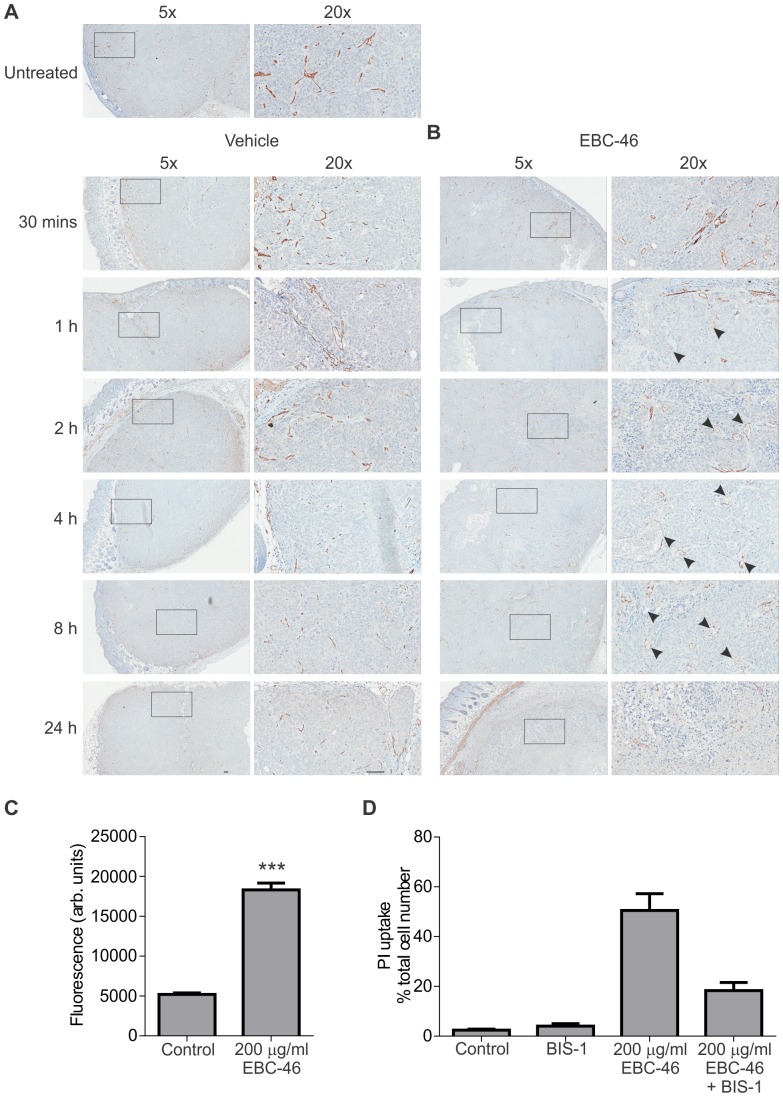
EBC-46 causes disruption and permeability of endothelial cells within tumor. **A**. Immunohistochemical analysis of CD31 staining of FaDu head and neck cancer tumors treated with EBC-46. FaDu tumors were allowed to reach 100 mm^3^ before they were treated with either vehicle (20% propylene glycol in water) or 50 nmol (30 µg) EBC-46, and harvested at the indicated times. Representative photomicrographs are shown of the tumor site. Black arrows – examples of vessels with compromised or disrupted structural integrity. Scale bars  = 100 µm. **B**. Monolayers of HUVEC cells were treated with 350 µM (200 µg/ml) EBC-46 for 30 mins, before being assessed for permeability to FITC labeled Dextran. (***, p = 0.0013; t-test). **C**. HUVEC cells were treated with 350 µM (200 µg/ml) EBC-46 for 30 mins with or without 5 µM bisindolylmaleimide-1, before being assessed by propidium iodide exclusion. Error bars – SD, n = 3.

It has been previously reported that activation of PKC, especially of PKC-β, can induce endothelial cell activation and permeability [30], [31]. Further, it was recently shown that PKC agonists can cause endothelial cell activation and permeability *in vivo* by disruption of structural integrity [29]. We therefore examined blood vessel integrity in FaDu tumors following EBC-46 treatment using detection of CD31 using immunohistochemistry. While treatment of FaDu tumors with vehicle alone led to no apparent disruption of vessel morphology, intra-lesional injection of EBC-46 resulted in the loss of structural integrity from as early as 30 min following treatment (Figure 6A). CD31 immunostaining showed vessels that were dilated and incomplete when compared to untreated tumors or tumors treated with vehicle alone (Figure 6A and B). Treatment of normal skin with EBC-46 led to less apparent damage, particularly of larger vessels, with the majority remaining structurally intact, although vessel swelling was evident (Figure S6A and B). We therefore examined the effect of EBC-46 on cultured monolayers of human umbilical vascular endothelial (HUVEC) cells. Monolayers of HUVEC cells were treated with 350 µM (200 µg/ml) EBC-46 (similar concentration to that present in the tumor; assuming 100 mm^3^ tumor volume and injection of 50 µl volume, a three-fold dilution of the initial 1,050 µM/600 µg/ml concentration) for 30 min, before being assessed for permeability using FITC-Dextran. The results show that short term, high dose treatment with EBC-46 leads to significantly increased permeability of the HUVEC cell monolayer (p = 0.0013, t-test; Figure 6C). Propidium iodide uptake experiments on HUVEC cells treated with EBC-46 showed that cells were compromised rapidly (5 min; data not shown). This uptake of propidium iodide by damaged HUVEC cells was PKC-dependent, as evidenced by inhibition with the pan-PKC inhibitor bisindolylmaleimide-1 (Figure 6D).

## Discussion

Activation of specific PKC isoforms in vascular endothelial cells, particularly the PKC-β isoforms, has previously been shown to induce permeability [30], [31]. Here we show that intra-lesional treatment with EBC-46, a novel PKC-activating compound with apparent specificity for PKC-β isoforms, induces permeability of endothelial cell monolayers *in vitro*, as well as vascular swelling and apparent disruption of vessel morphology *in vivo*. Further, sub-cutaneous injection of EBC-46 into normal skin led to significant levels of the drug found in the peripheral circulation. In contrast, intra-lesional injection of EBC-46 resulted in greatly reduced levels detected in the peripheral blood of mice. Additionally, the erythema and oedema observed following EBC-46 administration was significantly higher in mice with tumors compared to mice with normal skin. These results suggest a specificity of vascular damage within tumor sites compared to normal skin leading to anti-cancer efficacy. This specificity may reflect the disorganization and inherent “leakiness” of the vasculature within a solid tumor [32]. We hypothesize that the damage to the tumor vasculature prevents EBC-46 from entering the circulation. There is also a direct effect on tumor cell survival, as no viable tumor cells were evident four hours after injection by *ex vivo* culture. This is supported by *in vitro* data which showed treatment with EBC-46 was capable of inhibiting cell survival.

Previous studies elegantly showed that another PKC-activating ingenol ester, Ing3A (ingenol 3-angelate, ingenol mebutate, also known as PEP005) was able to penetrate deeper into the dermis following topical application compared to PMA, as it is a substrate for MDR1/P-glycoprotein (P-gp)/ABCB1 [29]. The authors showed that penetration of PMA after topical application was restricted to the epidermis of the skin, thereby sparing the vasculature in the sub-epidermal compartments and resulting in a lack of anti-tumor activity. Further, Li and colleagues also demonstrated that Ing3A bound to and inhibited P-gp whereas PMA did not [29]. Our results presented here additionally show that direct intra-tumoral injection of PMA only led to a transient reduction of tumor growth followed by a rapid relapse. In contrast, intra-tumoral injection of EBC-46 had an enduring anti-tumor effect.

We were unable to definitively demonstrate that neutrophils contribute significantly to the anti-tumor efficacy of EBC-46 by intra-lesional injection. Our conclusion was that neutrophils play a minor role in overall efficacy of EBC-46 from the experiments presented in this study. This is in contrast with previous work with other PKC activators (Ing3A/PEP005), where neutrophils were required for anti-tumor efficacy [15], [29]. However, the method of delivery used in this study (intra-tumoral injection) is very different compared to that used in the previous studies with the PEP005 (topical application). Further, the anti-neutrophil antibody used here was from a different, more neutrophil-specific hybridoma clone [33] than that used in previous studies. Nevertheless, in the current study approximately 6% of the cells in the peripheral WBC counts were found to be neutrophils.

It is important to note that intra-lesional injection of the prototypic PKC-activator PMA did not lead to a cure of tumors in the current study, but rather an initial shrinking of the tumor followed by a rapid relapse. In contrast, treatment with EBC-46 led to a rapid ablation of the tumor. In greater than 70% of cases, the response and cure was enduring and long term, as demonstrated by the lack of relapse of MM649 tumors over a period of 12 months. PKC activation must clearly play an important role in the action of EBC-46, since pre-treatment with the wide-spectrum PKC inhibitor bisindolylmaleimide-1 resulted in a loss of efficacy *in vivo*, and prevented endothelial cell dye uptake *in vitro*. However, we cannot rule out other known targets of diacylglycerol analogues other than PKC isoforms. Recent work has identified that Ing3A/PEP005 binds to and activates members of the RasGRP family of Ras activators, and that this activation may be in part responsible for the anti-cancer activity of the compound [34]. Future studies will investigate if EBC-46 similarly activates molecules in addition to PKC isoforms. In summary, we have identified and characterized EBC-46 as a novel PKC-activating compound. We demonstrate that a single intra-lesional bolus injection is sufficient for the short term regression and ultimate cure of multiple different cancer types in pre-clinical models. EBC-46 is currently being prepared under GMP conditions for use in an upcoming Phase I clinical trial.

## Supporting Information

Figure S1 **Translocation of PKC isoforms induced by EBC-46 in SK-MEL-28 cells.** PKC-EGFP isoform translocation in transiently transfected SK-MEL-28 cells following 1 h treatment with either 175 nM (100 ng/ml) PMA or EBC-46. Data was obtained from assessment of at least 50 cells per well from each of triplicate transient transfection experiments. Error bars - standard deviation. a – no data available due to mitochondrial location prior to and after treatment; b – no data available as isoform was toxic to SK-MEL-28 cells.
(TIF)

Figure S2 **Cell survival assays following treatment with EBC-46.** Dose response for cell killing by EBC-46 compared to PMA. Cells were treated with the indicated doses of either EBC-46 (blue) or PMA (red) for 4 days, before assay for cell survival using the sulforhodamine B assay. Data shown are mean ± SD from triplicate readings from three independent experiments, n = 3.
(TIF)

Figure S3 **Treatment of FaDu tumors with 30 µg EBC-46 or vehicle alone.** 2×10^6^ FaDu tumor cells were injected were injected (two tumors per mouse) on the hindquarter of 5 week old immuno-compromised BALB/c *Foxn1^nu^* mice. When the tumors had reached approximately 100 mm^3^, mice in the control group were treated with vehicle (20% propylene glycol in water, 50 µl) and the treatment group received 50 nmol (30 µg) EBC-46 in vehicle via a single intra-tumoral injection. Figure shows tumor appearance prior to treatment, 1 h following treatment, and 2, 5 and 11 days post treatment of tumors treated with vehicle alone (left) or 50 nmol (30 µg) EBC-46 (right). Also shown are ablated tumors 21 days following treatment with 50 nmol (30 µg) EBC-46. No vehicle only control tumors are shown due to the animals being euthanized at day 12 following treatment due to excessive tumor volume.
(TIF)

Figure S4 **EBC-46 Efficacy against head and neck or colon cancer tumors.** **A**. Tumor volume of FaDu HNSCC line in BALB/c *Foxn1^nu^* mice. **B**. Kaplan Meier plot of HT-29 tumor volume reaching greater than 100 mm^3^ in BALB/c *Foxn1^nu^* mice. **C**. Kaplan Meier plot of MC-38 tumor volume reaching greater than 100 mm^3^ in C57BL/6J mice. Grey - vehicle (20% propylene glycol in water); Black – 50 nmol (30 µg) EBC-46 (in vehicle).
(TIF)

Figure S5 **Effect of EBC-46 on normal skin.** Normal skin of BALB/c *Foxn1^nu^* mice was treated with either **A**. 50 µl vehicle (20% propylene glycol in water) or **B**. 50 nmol (30 µg) EBC-46 in vehicle. Arrows indicate examples of dilated blood vessels. Scale bar  = 100 µm.
(TIF)

Figure S6 **Effect of EBC-46 on normal skin vasculature.** Normal skin of BALB/c *Foxn1^nu^* mice was treated with either **A**. 50 µl vehicle (20% propylene glycol in water) or **B**. 50 nmol (30 µg) EBC-46 in vehicle. Arrows indicate examples of intact blood vessels. Scale bar  = 100 µm.
(TIF)
