# Supplementary figures and images for: Intra-Lesional Injection of the Novel PKC Activator EBC-46 Rapidly Ablates Tumors in Mouse Models
Source: PLoS One. 2014 Oct 1;9(10):e108887. doi: 10.1371/journal.pone.0108887 (PMC4182759; doi:10.1371/journal.pone.0108887)

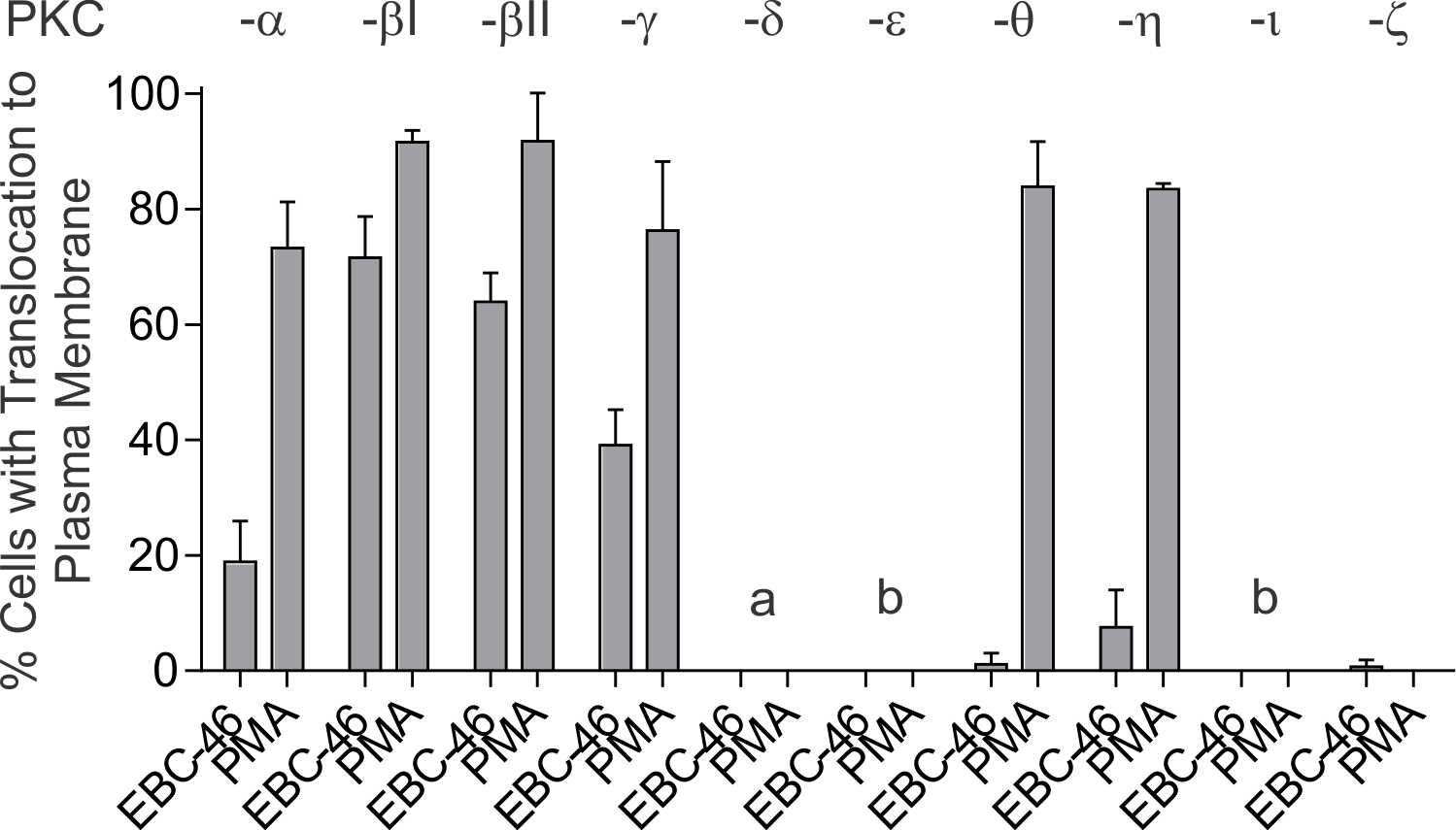

Supplement: Figure S1 — Translocation of PKC isoforms induced by EBC-46 in SK-MEL-28 cells. PKC-EGFP isoform translocation in transiently transfected SK-MEL-28 cells following 1 h treatment with either 175 nM (100 ng/ml) PMA or EBC-46. Data was obtained from assessment of at least 50 cells per well from each of triplicate transient transfection experiments. Error bars - standard deviation. a – no data available due to mitochondrial location prior to and after treatment; b – no data available as isoform was toxic to SK-MEL-28 cells. (TIF) [file pone.0108887.s001.tif]

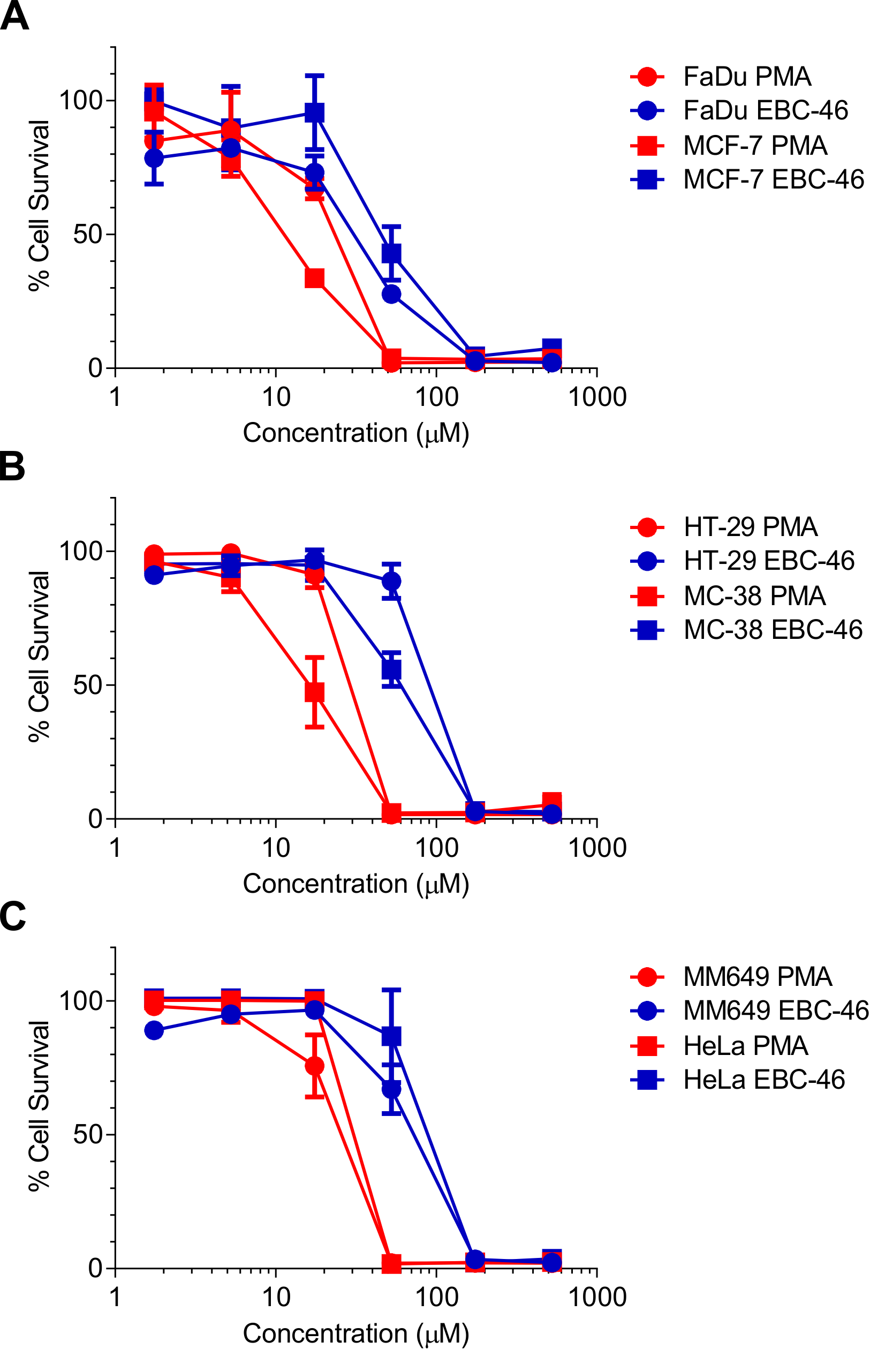

Supplement: Figure S2 — Cell survival assays following treatment with EBC-46. Dose response for cell killing by EBC-46 compared to PMA. Cells were treated with the indicated doses of either EBC-46 (blue) or PMA (red) for 4 days, before assay for cell survival using the sulforhodamine B assay. Data shown are mean ± SD from triplicate readings from three independent experiments, n = 3. (TIF) [file pone.0108887.s002.tif]

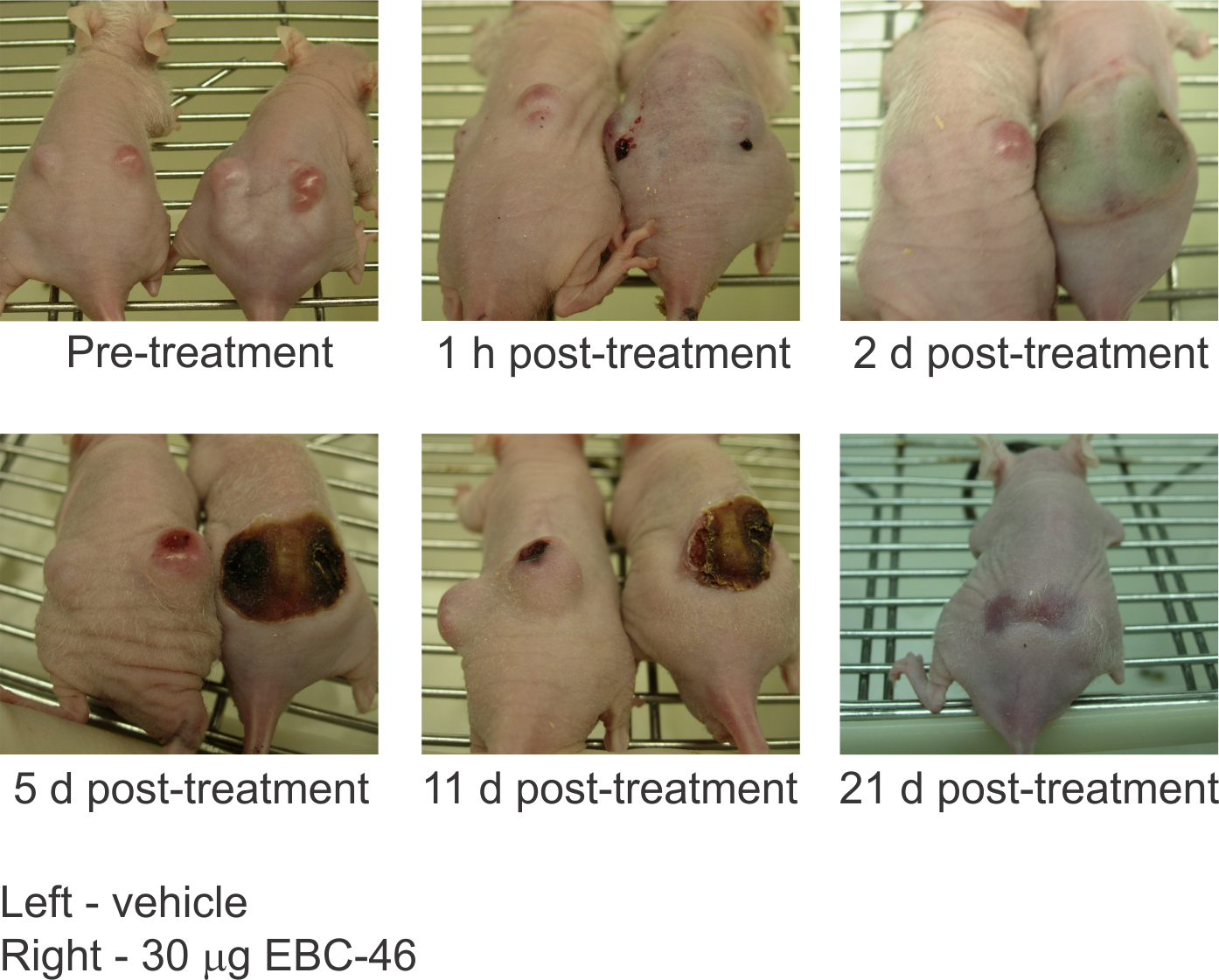

Supplement: Figure S3 — Treatment of FaDu tumors with 30 µg EBC-46 or vehicle alone. 2×10^6^ FaDu tumor cells were injected were injected (two tumors per mouse) on the hindquarter of 5 week old immuno-compromised BALB/c Foxn1nu mice. When the tumors had reached approximately 100 mm3, mice in the control group were treated with vehicle (20% propylene glycol in water, 50 µl) and the treatment group received 50 nmol (30 µg) EBC-46 in vehicle via a single intra-tumoral injection. Figure shows tumor appearance prior to treatment, 1 h following treatment, and 2, 5 and 11 days post treatment of tumors treated with vehicle alone (left) or 50 nmol (30 µg) EBC-46 (right). Also shown are ablated tumors 21 days following treatment with 50 nmol (30 µg) EBC-46. No vehicle only control tumors are shown due to the animals being euthanized at day 12 following treatment due to excessive tumor volume. (TIF) [file pone.0108887.s003.tif]

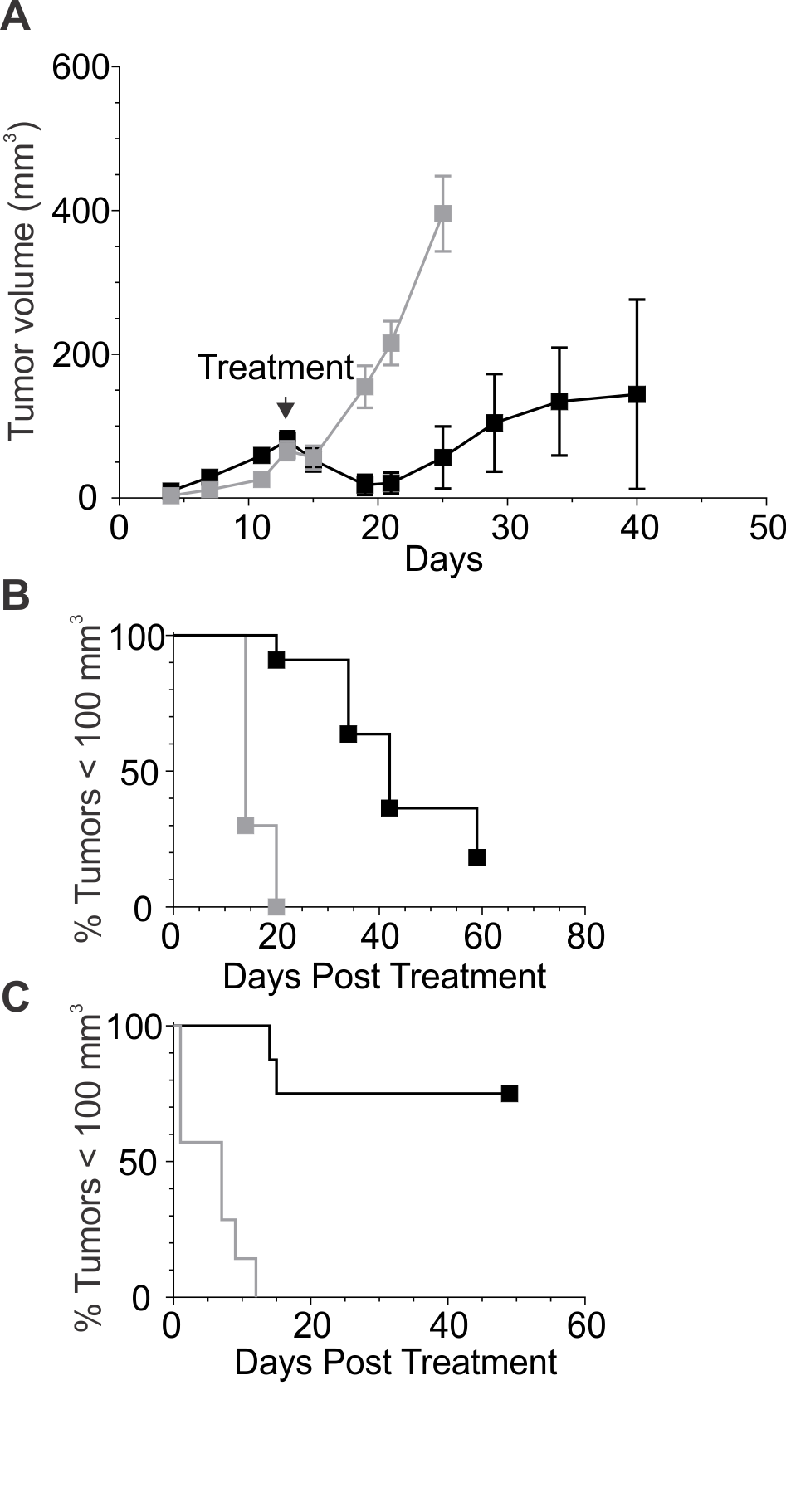

Supplement: Figure S4 — EBC-46 Efficacy against head and neck or colon cancer tumors. A. Tumor volume of FaDu HNSCC line in BALB/c Foxn1nu mice. B. Kaplan Meier plot of HT-29 tumor volume reaching greater than 100 mm3 in BALB/c Foxn1nu mice. C. Kaplan Meier plot of MC-38 tumor volume reaching greater than 100 mm3 in C57BL/6J mice. Grey - vehicle (20% propylene glycol in water); Black – 50 nmol (30 µg) EBC-46 (in vehicle). (TIF) [file pone.0108887.s004.tif]

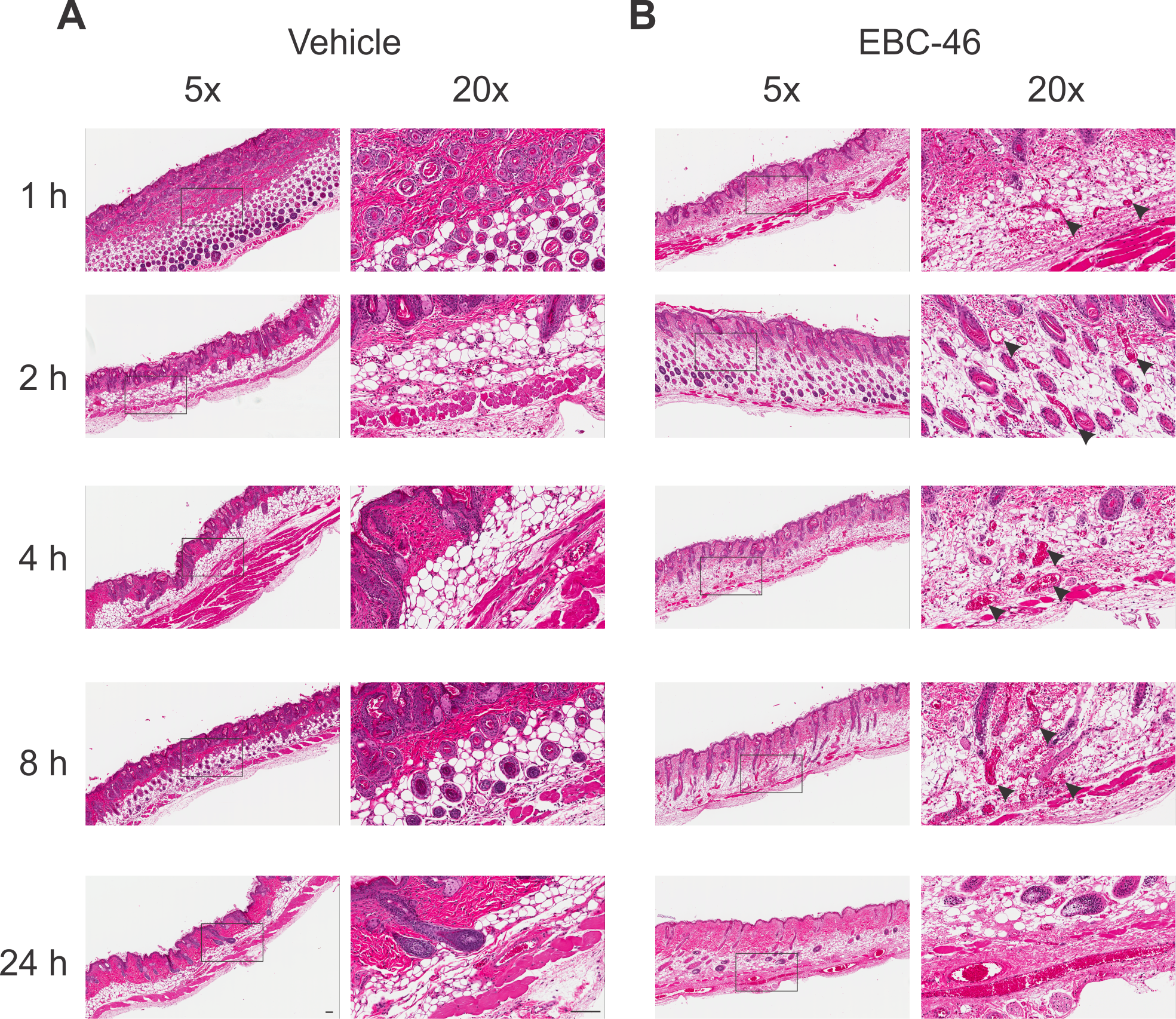

Supplement: Figure S5 — Effect of EBC-46 on normal skin. Normal skin of BALB/c Foxn1nu mice was treated with either A. 50 µl vehicle (20% propylene glycol in water) or B. 50 nmol (30 µg) EBC-46 in vehicle. Arrows indicate examples of dilated blood vessels. Scale bar = 100 µm. (TIF) [file pone.0108887.s005.tif]

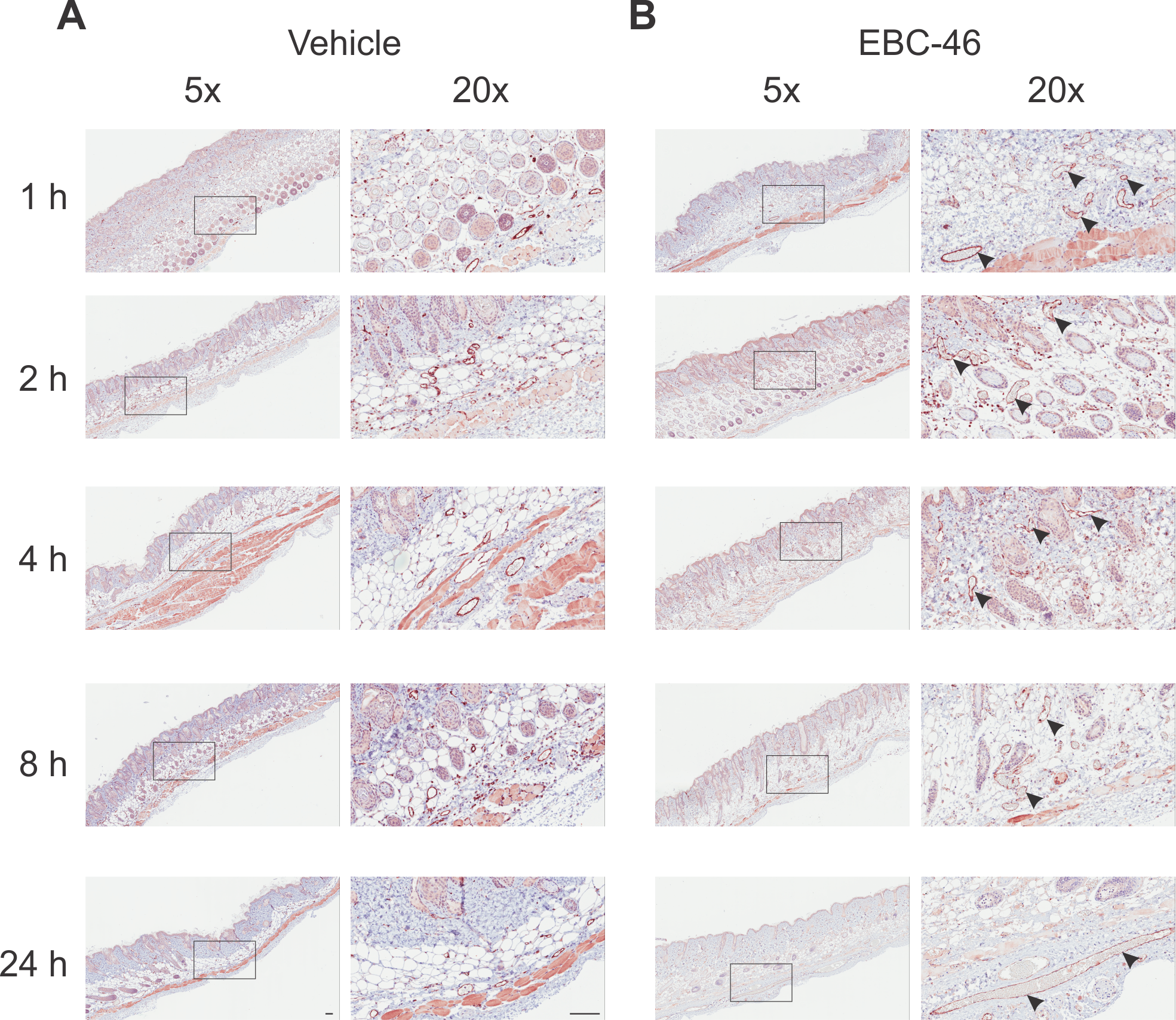

Supplement: Figure S6 — Effect of EBC-46 on normal skin vasculature. Normal skin of BALB/c Foxn1nu mice was treated with either A. 50 µl vehicle (20% propylene glycol in water) or B. 50 nmol (30 µg) EBC-46 in vehicle. Arrows indicate examples of intact blood vessels. Scale bar = 100 µm. (TIF) [file pone.0108887.s006.tif]
